# Intravascular Imaging–Guided Percutaneous Coronary Intervention: A Critical Appraisal of Evidence and Clinical Practice: Short title:Intravascular Imaging–Guided Percutaneous Coronary Intervention

**DOI:** 10.31661/gmj.v15i.4274

**Published:** 2026-05-17

**Authors:** Mohammad Rostamzadeh, Negar Jafari, Abdolmohammad Ranjbar, Hamid Biglary, Shirin Alord

**Affiliations:** ^1^ Cardiovascular Research Center, Tabriz University of Medical Sciences, Tabriz, Iran; ^2^ Department of Cardiology, School of Medicine, Urmia University of Medical Sciences, Urmia, Iran; ^3^ Cardiovascular Research Center, Health Policy and Promotion Institute, Kermanshah University of Medical Sciences, Kermanshah, Iran

**Keywords:** IPercutaneous Coronary Intervention, Intravascular Ultrasound, Optical Coherence Tomography, Coronary Artery Disease, Stents

## Abstract

Intravascular imaging has emerged as a critical adjunct to percutaneous coronary intervention (PCI), providing detailed insights into coronary anatomy, plaque morphology, and stent optimization beyond conventional angiography. Despite advances in PCI, adverse outcomes such as stent thrombosis and restenosis persist, often related to suboptimal lesion assessment and stent deployment. This review critically evaluates contemporary evidence from randomized controlled trials, large registries, and meta-analyses comparing intravascular imaging–guided and angiography-guided PCI. Current data consistently demonstrate that imaging guidance, particularly with intravascular ultrasound (IVUS), improves procedural optimization and reduces adverse cardiovascular events, with the greatest benefit observed in complex coronary lesions. Optical coherence tomography (OCT) provides superior spatial resolution and enhances detection of stent-related complications, although its impact on clinical outcomes appears more context-dependent. While recent guidelines strongly endorse intravascular imaging in complex PCI, real-world adoption remains limited due to economic, procedural, and training-related barriers. Variation in study design and variability in imaging protocols further complicate interpretation of existing evidence. Intravascular imaging–guided PCI represents a transition toward precision-guided coronary revascularization. Future research should focus on standardized imaging strategies, long-term outcomes, and integration with emerging technologies to optimize patient selection and procedural outcomes.

## Introduction

Coronary artery disease (CAD) remains a leading cause of morbidity and mortality worldwide, despite substantial advances in preventive strategies and revascularization techniques [1]. Percutaneous coronary intervention (PCI) has evolved as a cornerstone in the management of both stable ischemic heart disease and acute coronary syndromes, offering effective restoration of coronary blood flow and symptomatic relief [2]. Over the past decades, improvements in stent technology, pharmacotherapy, and procedural techniques have significantly enhanced clinical outcomes [2]. Nevertheless, adverse events such as in-stent restenosis, stent thrombosis, and target lesion failure continue to pose important clinical challenges [3][4].

Traditionally, PCI has been guided by coronary angiography, which provides a two-dimensional luminogram of the vessel lumen [5]. While angiography remains indispensable in clinical practice, it has inherent limitations, including its inability to accurately characterize plaque morphology, assess vessel wall pathology, or precisely evaluate stent expansion and apposition [6][7]. These limitations may contribute to suboptimal procedural outcomes, particularly in complex lesions such as bifurcations, chronic total occlusions, and left main disease [8][9].

Intravascular imaging modalities, notably intravascular ultrasound (IVUS) and optical coherence tomography (OCT), have been developed to overcome these constraints by providing high-resolution, cross-sectional visualization of the coronary vessel [7][10]. IVUS enables deep tissue penetration and assessment of plaque burden and vessel dimensions, whereas OCT offers superior spatial resolution, allowing detailed evaluation of stent strut apposition and microstructural features [6][11]. These technologies facilitate more accurate lesion assessment, guide stent sizing and deployment, and enable immediate identification of procedural complications [12].

Growing evidence from randomized controlled trials and observational studies suggests that intravascular imaging-guided PCI may improve both procedural and long-term clinical outcomes compared with angiography-guided strategies alone [8][13][14]. However, the extent of benefit, optimal imaging modality, and cost-effectiveness remain subjects of ongoing debate [15][16]. Additionally, real-world adoption of these technologies varies considerably across institutions and healthcare systems [6].

Nevertheless, important uncertainties persist regarding the optimal integration of intravascular imaging into routine clinical practice. Furthermore, variability in real-world adoption and variation in clinical evidence complicate translation into routine practice.

This review provides a structured and critical synthesis of contemporary evidence on intravascular imaging-guided PCI, emphasizing randomized trials, comparative effectiveness between IVUS and OCT, and their evolving role in clinical decision-making.

## Search Strategy and Study Selection

A structured literature search was conducted using PubMed, Embase, and Cochrane Library databases for studies published between 2010 and 2025. Search terms included "intravascular ultrasound," "optical coherence tomography," "PCI," "imaging-guided PCI," and "randomized controlled trials."

Priority was given to randomized controlled trials, large observational studies, and meta-analyses evaluating clinical and procedural outcomes. Additional references were identified through manual review of bibliographies and recent guideline documents.

Given the narrative design of this review, formal quantitative synthesis and standardized risk-of-bias assessment were not performed. However, key studies were critically appraised with attention to methodological quality, patient selection, endpoint definitions, and applicability to contemporary clinical practice.

## Overview of Intravascular Imaging Modalities

**Table:** Table1. Comparison of Intravascular Imaging Modalities: IVUS vs OCT

**Feature**	**IVUS**	**OCT**
**Imaging Principle**	High-frequency sound waves [7]	Near-infrared light [11]
**Spatial Resolution**	~100-150 µm [7]	~10-20 µm [11][17]
**Tissue Penetration Depth**	Up to ~10 mm [6]	~1-2 mm [6][7]
**Visualization of Vessel Wall**	Excellent (full vessel, EEM visible) [6]	Limited (superficial layers only) [7]
**Plaque Characterization**	Good for plaque burden and distribution [7]	Excellent for microstructure (fibrous cap, lipid, thrombus) [17]
**Stent Assessment**	Adequate (expansion, symmetry) [12]	Superior (apposition, strut coverage, microcomplications) [11][19]
**Blood Clearance Required**	No [6]	Yes (contrast injection needed) [7]
**Contrast Load**	Minimal [6]	Increased [7]
**Suitability in Renal Impairment **	Preferred [7]	Less favorable [7]
**Use in Large Vessels (e.g., Left Main) **	Preferred [6]	Limited by penetration [6]
**Detection of Calcification**	Moderate [7]	High sensitivity (especially superficial calcium) [17]
**Procedural Complexity**	Lower [6]	Higher [7]
**Cost Considerations**	Moderate [16]	Moderate to high [16]
**Learning Curve**	Moderate [10]	Steeper due to image interpretation [10]
**Common Clinical Indications**	Left main disease, long lesions, diffuse disease [6]	Stent optimization, edge dissection, thrombus evaluation [12][11]

Intravascular imaging has become an essential adjunct to percutaneous coronary intervention (PCI), enabling detailed visualization of coronary artery structure beyond the limitations of conventional angiography [6][7]. By providing cross-sectional and high-resolution images of the vessel lumen and wall, these modalities allow for precise lesion characterization, accurate vessel sizing, and optimization of stent deployment [12]. Among available technologies, IVUS and OCT are the most widely used in contemporary clinical practice [10]. Table-1 summarizes the key differences between intravascular imaging modalities.

### Intravascular Ultrasound (IVUS)

IVUS uses high-frequency ultrasound waves to generate real-time cross-sectional images of coronary arteries [7]. Its relatively deep tissue penetration (up to 10 mm) enables comprehensive assessment of vessel dimensions, plaque burden, and external elastic membrane (EEM) boundaries [6]. This makes IVUS particularly valuable for determining appropriate stent size and evaluating diffuse atherosclerotic disease, especially in large vessels such as the left main coronary artery [12].

### Optical Coherence Tomography (OCT)

OCT is a light-based imaging modality that employs near-infrared light to produce high-resolution (10-20 µm) images of the coronary artery [11][17]. This superior resolution enables detailed visualization of plaque microstructure, including fibrous caps, lipid pools, and calcific nodules, as well as precise assessment of stent strut apposition and neointimal coverage [17][18].

OCT is particularly advantageous for identifying procedural complications such as edge dissections, tissue prolapse, and thrombus formation [12][19]. It also facilitates accurate evaluation of stent expansion at a microstructural level [11]. However, OCT has limited tissue penetration (1-2 mm) compared with IVUS, which may restrict its ability to assess overall plaque burden and vessel size in larger arteries [6][7]. Furthermore, OCT requires transient blood clearance using contrast injection, which may increase contrast load and procedural complexity, particularly in patients with renal impairment [7].

### Comparative Considerations

Rather than representing competing technologies, IVUS and OCT should be viewed as complementary modalities with distinct clinical strengths [6][10]. IVUS is particularly advantageous for vessel sizing and assessment of diffuse or large-vessel disease, whereas OCT provides superior near-field resolution for detailed evaluation of stent-vessel interactions and superficial plaque characteristics [6][11]. Optimal utilization depends on lesion complexity, clinical context, and operator expertise [13][14].

## Mechanisms and Rationale for Imaging- Guided PCI

Although coronary angiography remains the foundation of percutaneous coronary intervention (PCI), its two-dimensional representation of the vessel lumen provides limited insight into the underlying atherosclerotic process and procedural adequacy [5]. Angiographic assessment is inherently constrained in its ability to evaluate plaque composition, vessel wall architecture, and stent-vessel interactions [6][7]. As a result, angiography-guided PCI may lead to suboptimal decision-making, particularly in complex lesions, thereby contributing to adverse clinical outcomes [8][20].

### Pathophysiological Basis of Suboptimal PCI

Adverse events following PCI, including in-stent restenosis, stent thrombosis, and target lesion failure, are often mechanistically linked to inadequate stent deployment [4][3]. Key contributors include stent underexpansion, malapposition, edge dissections, and geographic miss [4][21]. Among these, stent underexpansion is consistently identified as one of the strongest predictors of both early and late adverse events [21][22]. Inadequate lesion preparation, unrecognized calcification, and inaccurate vessel sizing further exacerbate these risks [12].

Additionally, failure to fully characterize plaque morphology may result in inappropriate interventional strategies [7]. For example, heavily calcified lesions may require plaque modification techniques (e.g., atherectomy or lithotripsy), while lipid-rich plaques may be more prone to distal embolization and peri-procedural myocardial injury [17][18].

### Enhanced Lesion Assessment

Intravascular imaging provides detailed, cross-sectional visualization of the coronary artery, enabling more accurate assessment of lesion severity, plaque burden, and vessel dimensions [7][10]. By delineating the external elastic membrane and luminal contours, imaging modalities such as IVUS facilitate precise stent sizing, reducing the risk of under sizing or oversizing [6].

Moreover, high-resolution imaging, particularly with OCT, allows for characterization of plaque composition, including identification of fibrous, lipid-rich, and calcific components [11][17]. This information is critical in guiding lesion preparation strategies and anticipating procedural challenges [12]. For instance, detection of deep or superficial calcium can inform the need for adjunctive plaque modification to ensure optimal stent expansion [4].

### Optimization of Stent Deployment

A central advantage of imaging-guided PCI lies in its ability to optimize stent deployment [6][12]. Intravascular imaging enables real-time assessment of stent expansion, symmetry, and apposition relative to the vessel wall [21]. Operators can use predefined imaging criteria such as minimum stent area thresholds to guide post-dilation and achieve optimal expansion [12].

Evidence suggests that achieving adequate stent expansion significantly reduces the risk of restenosis and stent thrombosis [8][13]. Imaging also facilitates precise identification of landing zones, minimizing geographic miss and ensuring full lesion coverage [6]. This is particularly important in diffuse disease and complex anatomies, where angiographic guidance alone may be insufficient [9].

### Detection and Management of Procedural Complications

Intravascular imaging enhances the detection of procedural complications that may be angiographically silent or underestimated [7][12]. These include edge dissections, tissue prolapse, thrombus formation, and incomplete stent apposition [11][19]. Early identification allows for immediate corrective measures, such as additional stenting or balloon optimization, potentially preventing downstream adverse events [12].

OCT, with its superior spatial resolution, is particularly effective in identifying subtle abnormalities at the stent-vessel interface, while IVUS provides a broader assessment of vessel integrity and plaque distribution [6][11]. Together, these modalities contribute to a more comprehensive evaluation of procedural success [10].

### Integrated Mechanistic Rationale

Collectively, these mechanisms explain how imaging guidance reduces procedural uncertainty and improves outcomes by enabling more precise and individualized intervention strategies [6][7]. By addressing the key mechanistic drivers of PCI failure, namely inadequate lesion assessment and suboptimal stent deployment, intravascular imaging provides a pathway to improved clinical outcomes [8][20].

## Evidence from Randomized Controlled Trials and Meta-Analyses

The evidence base for intravascular imaging-guided PCI has expanded substantially, moving from early mechanistic and surrogate-endpoint studies toward large randomized trials powered for clinical outcomes [16][20]. Overall, contemporary data support the view that imaging guidance improves procedural optimization and may reduce adverse cardiovascular events, particularly in anatomically complex PCI [8][14]. In contrast, the strength of evidence differs by imaging modality, lesion subset, and clinical endpoint [15].

### IVUS-Guided PCI

Randomized trials have consistently shown that IVUS guidance improves stent implantation quality and clinical outcomes compared with angiography alone[8][13]. In the IVUS-XPL trial, patients undergoing drug-eluting stent implantation for long coronary lesions had lower rates of major adverse cardiac events with IVUS guidance, and this benefit persisted at 5 years [23].

The ULTIMATE trial further strengthened the evidence base by enrolling an all-comers PCI population [24]. At 3 years, IVUS-guided drug-eluting stent implantation was associated with significantly lower target vessel failure and stent thrombosis compared with angiography-guided PCI [24]. Importantly, outcomes were best among patients achieving predefined IVUS optimization criteria, supporting the concept that benefit depends not only on imaging use, but also on acting upon imaging findings [24].

Although trials such as IVUS-XPL and ULTIMATE demonstrated improved clinical outcomes with IVUS guidance, their findings are influenced by protocol-driven optimization criteria and high operator expertise, which may limit generalizability to routine clinical practice [23][24].

### OCT-Guided PCI

The ILUMIEN IV trial demonstrated that OCT-guided PCI resulted in a larger minimum stent area compared with angiography guidance and reduced procedural abnormalities such as malapposition and edge dissection [25]. However, the trial did not show a significant reduction in target-vessel failure at 2 years. This discrepancy likely reflects limited statistical power for clinical endpoints and underscores the challenge of translating procedural improvements into measurable long-term clinical benefit [25].

By contrast, the OCTOBER trial demonstrated that OCT-guided PCI reduced major adverse cardiac events at 2 years in patients with complex bifurcation lesions [26]. This finding is clinically important because bifurcation PCI is technically demanding and prone to geographic miss, suboptimal side-branch treatment, and stent underexpansion [9][26].

### Complex PCI and Contemporary Trials

The RENOVATE-COMPLEX-PCI trial provided major contemporary support for intravascular imaging in complex coronary disease [27][28]. In patients with prespecified complex coronary lesions, imaging-guided PCI using IVUS or OCT reduced the risk of a composite of cardiac death, target-vessel myocardial infarction, or clinically driven target-vessel revascularization compared with angiography-guided PCI [28].

These findings align with updated European guidelines that recommend IVUS or OCT guidance for PCI in anatomically complex lesions, particularly left main stem disease, true bifurcations, and long lesions, with a Class I, Level A recommendation [29].

### Meta-Analytic Evidence

Recent meta-analyses of randomized trials generally support imaging-guided PCI over angiography-guided PCI [8][16]. A 2023 BMJ systematic review and meta-analysis reported that intravascular imaging guidance was associated with reduced cardiac death and cardiovascular outcomes compared with angiography guidance[30].

A 2024 JAHA meta-analysis similarly found improved outcomes with intravascular imaging-guided PCI, although effect sizes varied across endpoints and imaging modalities [20][31]. Network analyses suggest that IVUS has the most consistent evidence for reducing repeat revascularization, whereas OCT may provide stronger procedural optimization but less uniform clinical outcome benefit across all populations [15][31].

### Critical Appraisal

Taken together, randomized and meta-analytic evidence supports intravascular imaging-guided PCI as superior to angiography alone in many clinical contexts, especially complex PCI [8][20]. The most robust outcome data exist for IVUS, particularly in long lesions and all-comers PCI cohorts [13]. OCT offers unmatched resolution and clear procedural advantages, but its clinical benefit appears more context-dependent, with stronger evidence in complex bifurcation PCI than in broader PCI populations [15].

A balanced interpretation is therefore warranted. Imaging guidance should not be viewed as a uniform intervention; its benefit depends on lesion complexity, operator expertise, imaging interpretation, and adherence to optimization criteria [6]. The current evidence most strongly supports routine imaging in complex PCI, while selective use may remain reasonable in simpler lesions where angiographic results are unequivocally optimal [16].

Meta-analyses consistently demonstrate a relative reduction in adverse cardiovascular events with imaging-guided PCI; however, effect sizes are generally modest and heterogeneity across studies remains significant [15][16]. Outcomes are often driven by IVUS-dominant datasets, while OCT-specific effects are more variable [8][13]. These findings underscore the importance of interpreting pooled analyses in the context of study design, patient selection, and imaging protocols [16].

## Comparative Effectiveness: IVUS vs OCT vs Angiography-Guided PCI

**Table:** Table2. Main Strengths and limitation of imaging modalities in PCI

**Strategy**	**Main Strengths **	**Main Limitations **	**Best-Supported Use **
Angiography-guided PCI	Widely available, fast, familiar, low additional cost [5]	Limited plaque and vessel-wall assessment; may miss underexpansion or malapposition [6][21]	Simple lesions with clear angiographic result [8]
IVUS-guided PCI	Deep penetration; vessel sizing; plaque burden; left main assessment [6][7]	Lower resolution than OCT [11]	Left main PCI, long lesions, diffuse disease, large vessels [8][13]
OCT-guided PCI	Highest resolution; excellent stent apposition and edge assessment [11][17]	Requires contrast; limited penetration [7]	Bifurcations, stent optimization, thrombus or dissection assessment [12][26]

Coronary angiography remains the procedural foundation of PCI, but its role is increasingly understood as incomplete rather than sufficient [5][6]. Angiography provides a two-dimensional lumen silhouette, whereas IVUS and OCT provide cross-sectional information on vessel size, plaque burden, calcium, stent expansion, and complications [7][10]. This difference is clinically relevant because many mechanisms of PCI failure especially stent under expansion, geographic miss, malposition, and edge dissection may be underestimated or missed by angiography alone [4][21].

Table-2 highlights the major strengths and limitations of imaging modalities. Compared with angiography-guided PCI, IVUS-guided PCI has the most mature evidence base [8][13]. Trials such as IVUS-XPL and ULTIMATE, together with later meta-analyses, suggest that IVUS guidance reduces target lesion failure, target vessel revascularization, and stent thrombosis, particularly in long lesions, left main disease, and other complex anatomies [16][23][24]. Its deeper tissue penetration makes it especially useful for vessel sizing, plaque burden assessment, and large-vessel PCI [6].

OCT-guided PCI offers superior spatial resolution and is particularly effective for identifying stent malposition, tissue protrusion, thrombus, edge dissection, and superficial calcium [11][19]. In ILUMIEN IV, OCT guidance improved minimum stent area compared with angiography, although it did not significantly reduce target-vessel failure at 2 years [25]. By contrast, OCT showed clearer clinical benefit in complex bifurcation PCI, as seen in OCTOBER, suggesting that its clinical value may be strongest when high-resolution anatomical detail changes procedural strategy [26].

Large contemporary trials support the broader concept that intravascular imaging guidance is superior to angiography alone in complex PCI [8][20]. RENOVATE-COMPLEX-PCI showed that IVUS- or OCT-guided PCI reduced the composite of cardiac death, target-vessel myocardial infarction, or clinically driven target-vessel revascularization compared with angiography-guided PCI in complex coronary lesions [27]. Recent network meta-analysis also found that both IVUS and OCT reduced target lesion failure and repeat revascularization compared with coronary angiography, with no consistently dominant modality across all endpoints [15][16].

### Critical Interpretation

The available evidence supports a context-dependent approach to imaging selection rather than a universal preference for one modality over another [15][16]. IVUS has broader and more consistent long-term outcome evidence, particularly in complex and large-vessel PCI [13][24]. OCT provides more detailed near-field visualization and may be preferred when precise stent-vessel interaction is central to decision-making [11][26]. Current European guidance reflects this evidence shift by recommending IVUS or OCT for anatomically complex PCI, particularly left main, true bifurcation, and long lesions [29]. Thus, comparative effectiveness is best understood as context-dependent: angiography remains necessary, IVUS is often favored for vessel-level planning, and OCT is strongest for high-resolution optimization [6][7]. In contemporary practice, the central question is less whether imaging should be used, and more which modality best matches the lesion, patient risk profile, and procedural objective [12].

## Clinical Practice Guidelines and Recommendations

**Table:** Table3. Practical Recommendations for Clinical Use

**Clinical Scenario **	**Preferred Role of Imaging **	**Rationale**
Left main PCI	Strongly recommended [29]	Accurate vessel sizing, plaque distribution, and stent expansion assessment [6]
Long lesions	Strongly recommended [8]	Reduces geographic miss and improves stent optimization [21]
True bifurcation lesions	Strongly recommended [26]	Helps guide side-branch strategy and detect malapposition or edge complications [11]
Calcified lesions	Recommended/strongly considered [17]	Defines calcium arc, thickness, and need for plaque modification [22]
Stent failure	Recommended [4]	Identifies underexpansion, neoatherosclerosis, fracture, or malapposition [11]
ACS with unclear culprit lesion	Considered valuable [5]	Helps identify rupture, erosion, thrombus, or SCAD [18]
Simple focal lesions	Selective use [16]	Benefit may be smaller when angiographic result is clearly optimal [8]

**Figure-1:**
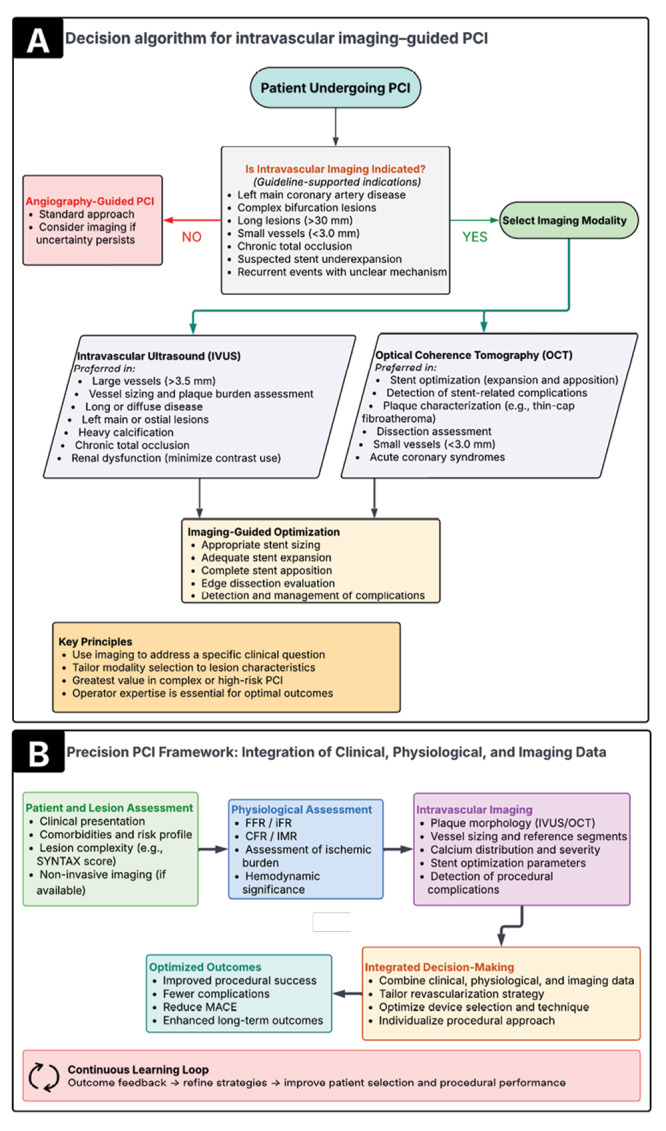
Figure 1. (A) Decision algorithm for the use of intravascular imaging during PCI, outlining guideline-supported indications for imaging and selection between IVUS and OCT.
(B) Precision PCI framework illustrating the integration of clinical assessment, physiological evaluation, and intravascular imaging to guide individualized revascularization strategies.

Table-3 summarizes the clinical recommendations and Figure 1 demonstrates the decision algorithm for intravascular imaging-guided PCI and precision PCI framework integrating clinical, physiological, and imaging data. Clinical guidelines increasingly recognize intravascular imaging as an important tool for improving the quality and safety of PCI [1][6]. Earlier recommendations generally supported selective use of IVUS or OCT, especially for left main PCI, stent failure, or uncertain angiographic findings [1]. More recent evidence from trials such as RENOVATE-COMPLEX-PCI, OCTOBER, ILUMIEN IV, ULTIMATE, and IVUS-XPL has strengthened the rationale for broader use in complex coronary intervention [23][24][25][26][27].

The 2024 ESC Guidelines for Chronic Coronary Syndromes provide one of the strongest contemporary endorsements [29]. They recommend intracoronary imaging guidance with IVUS or OCT when performing PCI in anatomically complex lesions, particularly left main stem disease, true bifurcations, and long lesions, with a Class I, Level A recommendation [29]. In acute coronary syndromes, ESC guidance also recognizes the value of IVUS and OCT for clarifying mechanisms such as plaque rupture, plaque erosion, thrombus, spontaneous coronary artery dissection, and ambiguous culprit lesions [5][18]. This is particularly relevant when angiography alone does not explain the clinical presentation or lesion morphology [7]. The 2021 ACC/AHA/SCAI Revascularization Guideline supported intravascular imaging as a reasonable strategy to guide PCI, particularly in left main or complex coronary anatomy [1]. More recent U.S. ACS guidance has further elevated the role of IVUS or OCT during PCI for left main or complex lesions, reflecting newer randomized evidence [15].

CFR = coronary flow reserve; CTO = chronic total occlusion; FFR = fractional flow reserve; iFR = instantaneous wave-free ratio; IMR = index of microcirculatory resistance; IVUS = intravascular ultrasound; MACE = major adverse cardiovascular events; OCT = optical coherence tomography; PCI = percutaneous coronary intervention.

## Critical Interpretation

Current guideline trends indicate a shift from viewing intravascular imaging as optional technology toward considering it a standard component of high-quality complex PCI [6][15]. However, recommendations remain most compelling for anatomically complex disease rather than uncomplicated lesions [16]. In practice, IVUS is often favored for left main, large-vessel, and diffuse disease because of its greater penetration, whereas OCT is particularly useful for bifurcations, stent-edge assessment, thrombus, and detailed evaluation of stent apposition [6][11].

Notably, the evolution of guideline recommendations reflects a shift from selective imaging use toward its recognition as a marker of procedural quality in complex PCI [7][12].

## Barriers to Adoption in Real-World Practice

Despite robust and growing evidence supporting intravascular imaging-guided PCI, its uptake in routine clinical practice remains inconsistent across regions and institutions [6][7]. Large registry data and observational studies indicate that the use of IVUS and OCT varies widely, with higher adoption in specialized centers and lower utilization in community settings [6]. This discrepancy reflects a multifactorial gap between evidence-based recommendations and real-world implementation [16].

### Economic and Reimbursement Constraints

One of the most significant barriers to widespread adoption is cost [16]. Intravascular imaging requires additional disposable catheters and may increase procedural expenses [6]. In healthcare systems with limited reimbursement or cost-containment pressures, operators and institutions may be less inclined to use imaging routinely [15]. Even in well-resourced settings, the perceived incremental cost particularly in cases where immediate benefit is not obvious can limit utilization [16].

Conversely, this perspective may not fully account for potential long-term cost savings associated with reduced rates of repeat revascularization and adverse events [8]. Economic analyses suggest that imaging-guided PCI may be cost-effective in high-risk or complex lesions, although cost-effectiveness remains context-dependent [15].

### Operator Expertise and Training

The effective use of intravascular imaging requires both technical proficiency and interpretative expertise [10]. While image acquisition is relatively straightforward, accurate interpretation of IVUS and especially OCT images involves a learning curve [11]. Misinterpretation may lead to inappropriate procedural decisions, potentially negating the benefits of imaging guidance [7].

Variability in training exposure during fellowship and limited access to structured educational programs further contribute to heterogeneity in operator confidence and utilization [6]. As a result, imaging is more frequently used by experienced interventional cardiologists or in high-volume centers [7].

### Procedural Time and Workflow Considerations

Intravascular imaging adds steps to the PCI procedure, including catheter preparation, image acquisition, and interpretation [12]. Although these steps are typically brief, they may be perceived as increasing procedural time, particularly in high-throughput catheterization laboratories [6].

In acute settings such as ST-elevation myocardial infarction (STEMI), concerns about delaying reperfusion may discourage routine imaging use, even though selective imaging can still be valuable in ambiguous or complex cases [5]. Workflow efficiency and team familiarity with imaging systems play a critical role in minimizing these barriers [12].

### Institutional and System-Level Factors

Adoption is also influenced by institutional culture, availability of imaging equipment, and support from healthcare systems [6]. Centers with established imaging protocols and leadership support tend to have higher utilization rates [7]. Conversely, limited access to imaging consoles, lack of standardized protocols, and absence of quality metrics tied to imaging use can hinder integration into routine practice [16].

Regional differences in guideline dissemination and healthcare infrastructure further contribute to variability [15]. For example, adoption rates tend to be higher in East Asia and parts of Europe compared with some regions of North America, reflecting differences in training, reimbursement, and clinical culture [6].

### Perception of Clinical Benefit

Another important barrier is the perception that intravascular imaging may not provide substantial incremental benefit in all cases [16]. While evidence strongly supports its use in complex PCI, the benefit in simple lesions is less pronounced [8]. This has led to selective rather than routine use, with some operators reserving imaging for challenging cases or when angiographic uncertainty exists [15].

Additionally, earlier trials focusing on surrogate endpoints rather than hard clinical outcomes may have contributed to skepticism, although more recent randomized trials have addressed this limitation [16].

### Strategies to Improve Adoption

Addressing these barriers requires a multifaceted approach [6]. Enhanced training programs and incorporation of intravascular imaging into interventional cardiology curricula can improve operator proficiency [10]. Standardized imaging protocols and optimization criteria may reduce variability and streamline workflow [12]. Furthermore, alignment of reimbursement policies with evidence-based practice could incentivize appropriate use [15].

Importantly, continued generation of high-quality evidence particularly cost-effectiveness analyses and real-world outcome data will be essential to reinforce the clinical and economic value of imaging-guided PCI [8][16].

Conversely, the underutilization of intravascular imaging may also reflect cognitive bias and reliance on angiographic familiarity, rather than purely economic or logistical constraints [16].

## Limitations of Current Evidence

Despite substantial progress in the evidence base supporting intravascular imaging-guided PCI, several important limitations constrain the interpretation and generalizability of current findings [15][16]. These limitations should be carefully considered when translating trial data into clinical practice [8].

### Heterogeneity in Study Design and Patient Populations

Randomized controlled trials evaluating imaging-guided PCI differ considerably in inclusion criteria, lesion complexity, and patient risk profiles [8][16]. Some studies, such as IVUS-XPL and OCTOBER, focus on specific high-risk lesion subsets (e.g., long lesions or bifurcations), whereas others, such as ULTIMATE, adopt an all-comers design [23][24][26]. This heterogeneity complicates cross-trial comparisons and limits the ability to derive uniform conclusions applicable to all PCI populations [15].

Furthermore, differences in stent platforms, adjunctive pharmacotherapy, and procedural techniques across trials introduce additional variability that may influence outcomes independently of imaging guidance [8].

### Endpoint Selection and Statistical Power

Many earlier studies of intravascular imaging focused on surrogate endpoints, such as minimum stent area, late lumen loss, or binary restenosis, rather than hard clinical outcomes [16]. Although more recent trials have incorporated endpoints such as target vessel failure and major adverse cardiovascular events (MACE), not all have been adequately powered to detect differences in relatively low-frequency outcomes such as cardiac death or stent thrombosis [15]. As a result, some trials demonstrate clear improvements in procedural metrics without statistically significant reductions in clinical events, raising questions about the direct translation of mechanistic benefits into long-term outcomes [13].

### Variability in Imaging Protocols and Operator Dependence

The benefit of imaging-guided PCI is closely linked to how imaging is used [6]. Across studies, there is considerable variability in imaging protocols, including criteria for stent optimization, thresholds for minimum stent area, and decision-making algorithms [12]. Trials such as ULTIMATE have shown that achieving predefined optimization criteria is associated with improved outcomes, underscoring the importance of protocol adherence [24]. In addition, intravascular imaging is inherently operator-dependent [10]. Differences in image acquisition, interpretation, and response to imaging findings can influence procedural decisions and outcomes [7]. This variability introduces potential bias and limits reproducibility across centers with differing levels of expertise [6].

### Limited Direct Comparisons Between IVUS and OCT

Although both IVUS and OCT have been compared with angiography-guided PCI, direct head-to-head randomized comparisons between the two modalities remain relatively limited [15]. Available studies and network meta-analyses suggest broadly comparable clinical outcomes, but they are often underpowered to detect modest differences [16]. Moreover, the distinct physical properties of IVUS and OCT make them inherently suited to different clinical scenarios, complicating efforts to establish superiority of one modality over the other [6]. As such, current evidence supports a complementary rather than competitive relationship between these technologies [7].

### Generalizability to Real-World Practice

Patients enrolled in randomized trials are often highly selected and treated in experienced, high-volume centers with standardized protocols [8]. Consequently, trial results may not fully reflect outcomes in routine clinical practice, where patient complexity, operator experience, and resource availability vary widely [16]. Real-world registry data suggest lower rates of imaging utilization and potentially different outcomes, highlighting a gap between trial efficacy and real-world effectiveness [6].

### Economic Considerations and Cost-Effectiveness

Although some studies suggest that imaging-guided PCI may be cost-effective—particularly in complex lesions—robust economic data remain limited [15]. Cost-effectiveness analyses are sensitive to assumptions regarding event reduction, device costs, and healthcare system structure [16]. The lack of standardized economic evaluations across diverse healthcare settings represents an important gap in the literature [8].

### Long-Term Outcomes and Emerging Technologies

While several trials now report follow-up beyond 2-3 years, truly long-term data (e.g., beyond 5-10 years) remain limited [23]. Furthermore, rapid technological advancements including high-definition IVUS, hybrid imaging systems, and artificial intelligence-assisted interpretation are not fully represented in existing trials [6]. As a result, current evidence may not fully capture the potential benefits of next-generation imaging technologies [12].

### Critical Perspective

These limitations underscore the need for cautious interpretation of current evidence [15][16]. Future studies should focus on standardized imaging protocols, adequately powered clinical endpoints, and broader inclusion of real-world populations to improve generalizability and clinical applicability [6][7].

## Future Directions and Emerging Technologies

The field of intravascular imaging-guided PCI is evolving rapidly, driven by the need for greater precision, improved clinical outcomes, and more efficient procedural workflows [6][12]. While current technologies such as IVUS and OCT have already demonstrated clinical value, emerging innovations aim to enhance image quality, reduce operator dependence, and integrate imaging with complementary diagnostic tools [7].

### Advances in Imaging Technology

Recent developments in high-definition intravascular ultrasound (HD-IVUS) have improved spatial resolution while preserving the deep tissue penetration characteristic of conventional IVUS [6]. This allows for more accurate delineation of plaque morphology and stent expansion without sacrificing vessel-level assessment [12]. Similarly, next-generation OCT systems are being developed to reduce contrast requirements and improve penetration depth [7]. Faster pullback speeds and enhanced image processing algorithms have improved procedural efficiency and reduced the risk of contrast-induced nephropathy, expanding the applicability of OCT in higher-risk populations [7][11].

### Hybrid and Multimodality Imaging

An important emerging concept is the integration of multiple imaging modalities into a single platform [6]. Hybrid systems combining IVUS and OCT or imaging with near-infrared spectroscopy (NIRS) offer complementary information on plaque burden, composition, and vulnerability [10]. These systems may enable more comprehensive lesion assessment, particularly in high-risk plaques where both structural and compositional information are clinically relevant [7]. The combination of anatomical imaging with physiological assessment tools, such as fractional flow reserve (FFR) and non-hyperemic pressure ratios (NHPRs), represents another promising direction [12]. This integrated approach allows clinicians to assess both the functional significance of a lesion and its structural characteristics, supporting more individualized revascularization strategies [6].

### Artificial Intelligence and Automation

Artificial intelligence (AI) and machine learning are increasingly being applied to intravascular imaging to assist with image interpretation, lesion classification, and procedural decision-making [6][12]. AI-driven algorithms can automatically identify key features such as lumen borders, plaque composition, calcium distribution, and stent expansion metrics [7]. These technologies have the potential to reduce inter-operator variability, shorten interpretation time, and standardize imaging-guided PCI [10]. In the future, real-time decision-support systems may provide automated recommendations for stent sizing, optimization targets, and need for adjunctive therapies, thereby enhancing procedural consistency and outcomes [12].

### Improved Workflow and Accessibility

Efforts are also underway to streamline imaging workflows and improve accessibility [6]. Advances in catheter design, software integration, and user interfaces aim to reduce procedural complexity and facilitate broader adoption in routine practice [7]. Simplified imaging protocols and standardized optimization criteria may further lower barriers to use [12]. In addition, expanding training programs and incorporating simulation-based education into interventional cardiology curricula will be essential to ensure that operators can effectively utilize these advanced technologies [10].

### Ongoing Trials and Research Priorities

Future clinical research will play a critical role in defining the next phase of imaging-guided PCI. Key priorities include:

• Large-scale randomized trials comparing IVUS and OCT directly in diverse patient populations

• Long-term outcome studies assessing durability of benefit beyond 5 years

• Cost-effectiveness analyses across different healthcare systems

• Evaluation of imaging-guided strategies in acute coronary syndromes and high-risk subsets

• Validation of AI-assisted imaging tools in prospective clinical settings

A key future direction is the development of a "precision PCI" framework integrating intravascular imaging, physiological assessment, and patient-specific risk stratification [12][6]. Such an approach may enable tailored revascularization strategies, moving beyond uniform procedural algorithms toward individualized decision-making supported by multimodal data and artificial intelligence [7][10].

### Forward-Looking Perspective

Intravascular imaging is transitioning from a supportive tool to a central component of precision interventional cardiology [6][7]. The integration of advanced imaging technologies, artificial intelligence, and physiological assessment is expected to refine procedural strategies, reduce variability, and improve patient outcomes [12]. As these innovations mature and are validated through rigorous clinical research, they have the potential to transform imaging-guided PCI from a specialized practice into a universal standard of care [15].

## Conclusion

Intravascular imaging has redefined the standard of care in contemporary PCI by enabling precise assessment of coronary anatomy and optimization of stent deployment. Current evidence supports its routine use in complex coronary lesions, with IVUS demonstrating the most consistent clinical benefit and OCT providing superior procedural detail in selected contexts.

Despite strong evidence and guideline endorsement, adoption remains variable due to economic, procedural, and educational barriers. Future advances in imaging technology, artificial intelligence, and integrated diagnostic approaches are expected to further enhance the role of imaging-guided PCI, supporting a transition toward precision-based coronary intervention. Ultimately, the integration of intravascular imaging into routine practice will depend on aligning clinical evidence, operator expertise, and healthcare system resources to achieve consistent and equitable implementation.

## Conflict of Interest

The authors declare no conflict of interest.

## AI Disclosure Statement

During the preparation of this manuscript, the authors used ChatGPT, OpenAI company for language editing, grammar improvement, and liboberry.com for reference management. After its use, the authors thoroughly reviewed, verified, and revised all AI-assisted content to ensure accuracy and originality. The authors take full responsibility for the integrity and final content of the published article.
